# Bioactive restorative materials in dentistry: a comprehensive review of mechanisms, clinical applications, and future directions

**DOI:** 10.1007/s10266-025-01162-w

**Published:** 2025-08-16

**Authors:** Dina Abozaid, Amr Azab, Mohammad A. Bahnsawy, Mohamed Eldebawy, Abdullah Ayad, Romesa soomro, Enas Elwakeel, Maged Ahmed Mohamed

**Affiliations:** 1 Dental Biomaterials Department, Faculty of Dentistry, Tanta University, El-Geish Street, Tanta, 31511 Egypt; 2 Prosthodontics Department, Faculty of Dentistry, Tanta University, Tanta, Egypt; 3 Faculty of Dental Medicine, Al-Azhar University, Cairo, Egypt; 4 Faculty of Dentistry, Tanta University, Tanta, Egypt; 5 College of Dentistry, University of Basrah, Basrah, Iraq; 6 Molecular Genetics and Proteomics Department, Bayan National Lab for Advanced Medical Diagnostics, Basrah, Iraq; 7 Physics Department, Faculty of Science, Universiti Putra Malaysia, 43300 Selangor, Malaysia; 8 Medical Research Group of Egypt, Cairo, Egypt

**Keywords:** Bioactive, Direct restorative materials, Composite resin, Glass ionomer, Ion-releasing restorations

## Abstract

Bioactive restorative materials represent a paradigm shift in modern dentistry, moving from passive restorations to functional materials that actively promote oral health. This review comprehensively explores the evolution, mechanisms of action, methods of assessment, and clinical applications of direct bioactive restorative materials, including glass ionomer cements (GICs), resin-based composites, and ion-releasing materials. This review critically analyzes their roles in remineralization, fluoride release, antimicrobial activity, and tissue regeneration, while also addressing their mechanical properties and limitations. A systematic literature search was conducted across major databases, including PubMed, Scopus, Web of Science, and ScienceDirect. Key findings reveal that bioactive GICs, resin-based composites, and ion-releasing materials exhibit varying degrees of bioactivity through ion release, apatite layer formation, and collagen interaction. Recent advances in calcium phosphate-based fillers, bioactive glass additives, and antimicrobial agents have significantly improved these materials’ therapeutic potential. However, challenges remain regarding mechanical strength, long-term stability, and standardization of bioactivity assessment. Future research should focus on developing standardized testing protocols, optimizing mechanical performance, and conducting rigorous long-term clinical trials to fully harness the potential of bioactive restorative materials in dental practice. Also identifying the key knowledge gaps and proposing future research directions to advance the field.

## Introduction

Dental materials have undergone a significant transformation over the past few decades, evolving from passive, biologically inert substances to bioactive materials designed to actively interact with the oral environment. Historically, dental restorative materials primarily focused on restoring form and function, with minimal consideration for their biological impact. However, recent breakthroughs in material science have led to the development of bioactive substances that elicit beneficial biological outcomes, including tissue repair, remineralization, and antimicrobial action [1–4].

The concept of bioactivity in dental materials is rooted in their ability to interact with biological tissues, inducing positive cellular responses and promoting healing. Bioactive materials are broadly defined as those capable of releasing ions, forming apatite-like layers, and stimulating cellular activity, all of which contribute to improved clinical outcomes. However, despite the growing interest in bioactive materials, a consensus on the precise definition and criteria for bioactivity remains elusive, particularly in restorative dentistry [5]. This ambiguity hinders the standardization of testing methods and the interpretation of clinical results [6, 7].

This review aims to provide a comprehensive and critical overview of bioactive restorative dental materials, focusing on their mechanisms of action, clinical applications, and assessment methods. In addition, the challenges and future directions in the development of bioactive materials, with an emphasis on the need for standardized criteria to evaluate their efficacy, safety, and long-term clinical performance will be discussed. The existing controversies and knowledge gaps surrounding the clinical translation of these materials will be addressed.

## Mechanisms of bioactivity

The term “bioactive” refers to materials that exert a biological effect on surrounding tissues, typically promoting repair, regeneration, or modulation of bacterial activity. In restorative dentistry, bioactive materials are expected to not only restore lost tooth structure but also actively stimulate cellular responses or control microbial interactions [8, 9]. However, the definition of bioactivity remains contentious, with some researchers arguing that no current dental restorative material fully meets the criteria for bioactivity. It’s crucial to differentiate between materials exhibiting “bioactivity” and those simply being “biocompatible.”

The 2023 FDI Policy Statement provides a framework for defining bioactive dental materials, categorizing them based on their mechanisms of action (biological, mixed, or chemical) and outlining essential criteria for their use. These criteria include clear mechanisms of action, documented bioactive effects, duration of activity, absence of adverse side effects, and preservation of the material’s primary restorative function [10]. However, the practical application of these criteria in evaluating different materials needs further scrutiny.

Bioactive restorative materials in dentistry are designed to interact beneficially with dental tissues, promoting processes such as remineralization and tissue repair. The bioactivity of these materials can be attributed to several mechanisms:

### Ion release and precipitation

Bioactive materials such as 45S5 bioactive glass rapidly liberate calcium, phosphate, sodium and soluble silica ions into the surrounding fluid [1, 10]. The resulting ionic burst elevates the degree of supersaturation, so an amorphous calcium-phosphate (ACP) precursor nucleates on the material surface. Bakry et al. showed that this transient brushite layer transforms within 24 h into a hydroxyapatite coating enriched with silica, which infiltrates demineralized enamel and restores its micro-hardness [11]. Consequently, the kinetics of ion release and subsequent mineral precipitation are pivotal determinants of the long-term remineralization performance of these materials.

### Formation of apatite layer

When 45S5 bioactive glass contacts an acidic aqueous phase it liberates Ca^2^⁺, PO₄^3^⁻ and soluble silica; these ions quickly supersaturate the fluid, precipitating an amorphous calcium-phosphate (ACP) film that densifies through brushite and monetite intermediates and ultimately matures toward hydroxyapatite [1, 12]. Bakry et al. demonstrated that the resulting interaction layer spreads over the entire dentin surface, penetrates 3–5 µm into the tubules and raises nano-hardness and elastic modulus within 24 h [13]. A subsequent durability study by the same group showed that this layer withstands 6000 simulated brushing strokes, maintains a low fluid conductance and outperforms oxalate-based desensitizers in keeping tubules sealed. Because the phase composition evolves over time, the final apatite’s crystallinity and acid tolerance are influenced by both the glass chemistry and the oral milieu, highlighting ion release kinetics as a key design parameter [14].

### Interaction with collagen fibers

Bioactive materials can induce mineral precipitation between collagen fibers, enhancing the structural integrity of the dentin matrix. This interaction also helps in reducing enzymatic degradation by matrix metalloproteinases (MMPs), which are involved in the breakdown of collagen [1]. The long-term stability of this collagen-mineral interaction and its impact on the durability of the restoration require further investigation.

### Mixed biological and chemical mechanisms

Some materials, like those containing calcium hydroxide, exhibit both chemical and biological effects. They can induce the formation of tertiary dentin by activating signaling molecules within the dentin, while also providing a chemical environment conducive to mineralization [10]. The specific signaling pathways involved in tertiary dentin formation and their modulation by bioactive materials are areas of active research.

### Antimicrobial effects

Bioactive materials may incorporate antimicrobial agents or release ions that inhibit bacterial growth, further supporting oral health by preventing caries and other infections [10]. The spectrum of antimicrobial activity, the potential for developing bacterial resistance, and the long-term effects on the oral microbiome need careful consideration [15]. These mechanisms highlight how bioactive restorative materials can enhance dental health by promoting tissue repair and preventing disease progression. However, the relative contribution of each mechanism and its synergistic effects remains to be fully elucidated.

## Classification of bioactive restorative materials

Bioactive restorative materials and bioactive glasses represent a diverse and expanding group of substances with varying compositions, mechanisms of action, and degrees of bioactivity. For clarity and to facilitate comparison, these materials can be classified according to two main criteria: composition and degree of bioactivity.

### Classification by composition

Bioactive materials can be grouped based on their major chemical constituents:

#### Silica-containing bioactive glasses

The prototypical example is Bioglass® 45S5, composed primarily of SiO₂, Na₂O, CaO, and P₂O₅. These glasses are well-known for their ability to bond to hard and soft tissues and to form a hydroxyapatite layer when exposed to physiological fluids [16].

#### Silica-free bioactive glasses

Some newer formulations exclude silica, instead incorporating other oxides or phosphates to tailor bioactivity and mechanical properties [17].

#### Calcium phosphate-based materials

This group includes synthetic hydroxyapatite, β-tricalcium phosphate, and calcium phosphate cement. These materials are valued for their chemical similarity to the mineral phase of bone and teeth, and their ability to promote remineralization [18].

#### Resin-based composites with bioactive fillers

These materials combine a resin matrix with bioactive glass or calcium phosphate particles, aiming to provide both mechanical strength and therapeutic ion release [19].

#### Glass ionomer cements (GICs)

While not traditionally classified as bioactive glasses, GICs exhibit bioactivity through fluoride and ion release, contributing to remineralization and antibacterial effects [20–22].

### Classification by degree of bioactivity

The degree of bioactivity reflects a material’s ability to induce a biological response, typically categorized as:

#### High bioactivity

Materials that can rapidly form a direct bond with living tissues via the precipitation of an apatite layer. Classic examples include Bioglass® 45S5 and certain calcium phosphate cements [16].

#### Moderate bioactivity

Materials that induce slower or less robust apatite formation, or require specific conditions (e.g., acidic or basic environments) to exhibit bioactivity. Some resin-modified glass ionomers and newer bioactive composites fall in this category [19].

#### Low bioactivity

Materials that release beneficial ions but do not readily form an apatite layer or bond directly to tissue. Some glass ionomer cement, and ion-releasing resins may be considered low bioactivity materials [20].

## Mechanism of bioactivity

The concept of bioactivity originally referred to specialty glasses that elicit a positive biological response when introduced into the human body [23]. In modern usage, the term refers to several essential features: the emission of ions that benefit biological systems, the formation of a calcium phosphate layer, and the promotion of cell differentiation and proliferation [5]. It is crucial to understand, however, that merely releasing ions does not qualify a material as bioactive; the ion release must occur alongside the other two processes [10]. Moreover, in certain materials—such as specific polymers—bioactivity may also include the release of biological signaling molecules that support tissue restoration and repair. [5]. This aspect was not part of the original definition of bioactive glasses when the term was first applied. A critical analysis of the evolution of the bioactivity concept and its implications for restorative materials is warranted.

## The evolution of bioactivity

The development of bioactive materials in dentistry can be traced back to 1969, when Professor Larry Hench and his team introduced Bioglass [24]. As early as 1971, research articles described this material as bioactive, emphasizing its unique ability to create a mechanically robust bond between host tissue and implant [25]. The inaugural bioactive glass, Bioglass 45S5, is composed of four primary ingredients: SiO₂ (46.1% by mass), CaO (26.9%), Na₂O (24.4%), and P₂O₅ (2.6%) [26, 27]. Its extensive application in bone-contact procedures is largely due to its capacity to develop a hydroxyapatite carbonate layer when immersed in simulated body fluid [28]. In living systems, Bioglass interacts closely with bone cells—especially osteoblasts—which are activated by the initial deposition of a calcium phosphate layer, leading to a firm bond between the glass and emerging bone [26, 27, 29].

Following the original composition, a variety of bioactive glasses have been produced and their biological actions investigated in depth. Most new formulations have utilized the SiO₂–CaO–Na₂O–P₂O₅ framework, though other glass variants containing five or more components have also been explored [30]. One example is the apatite-wollastonite (A/W) glass ceramic, which bonds with bone and is marketed for clinical use as Cerabone® A-W [31].

In addition, synthetic hydroxyapatite and β-tricalcium phosphate [32], also demonstrate the capability to bond with living bone in a manner similar to Bioglass [18]. These substances trigger analogous surface reactions: they release calcium and phosphate ions into the surrounding body fluid, leading to the precipitation of an amorphous calcium phosphate layer that osteoblasts colonize in vivo, ultimately giving rise to fully functional natural bone [33, 34].

Furthermore, calcium phosphate cements formed in situ are recognized as bioactive as well [35]. These cements are produced by mixing aqueous slurries of tetra-calcium phosphate and di-calcium phosphate, which react to form hydroxyapatite [36]. The hydroxyapatite then precipitates as a solid mass that retains all the water from the original slurry [36]. Resulting in a material that, although relatively weak, is biologically active and capable of directly bonding with bone. This makes it clinically useful for repairing craniofacial defects and bone fractures [37, 38].

## Assessment of bioactivity

Both in vitro and in vivo methods are employed to characterize the bioactivity of these materials. However, a standardized approach to bioactivity assessment is lacking, which complicates the comparison of results across different studies.

### Apatite formation

#### In vitro assessment using simulated body fluid (SBF)

In vitro assessments often involve immersing the samples in SBF to simulate physiological conditions. This method allows researchers to evaluate the material’s bioactivity and its ability to form an apatite layer, which is indicative of its potential to integrate with biological tissues. The specimens are typically soaked in SBF for a specified duration, often ranging from several days to weeks, while maintaining a physiological pH (around 7.4) [39]. Simulated body fluid (SBF) is extensively employed in bioactivity assessments as an in vitro screening test to identify materials that exhibit bioactivity in vivo. This method involves exposing materials to SBF, which mimics the composition of human plasma, particularly in terms of key ions like Ca^2^⁺ and PO₄^3^⁻ [40, 41]. The primary purpose of using SBF is to assess the material’s ability to form hydroxyapatite (or hydroxy-carbonate apatite), which is indicative of bioactivity [40, 41].

##### Purpose of SBF testing

The main goal of SBF testing is to evaluate the material’s ability to induce apatite formation on its surface. This process is crucial for assessing bioactivity, as it suggests the material can integrate with bone tissue by forming a bone-like apatite layer [40, 41].

While ion release is an important aspect of bioactivity, SBF testing primarily focuses on apatite formation as a direct indicator of bioactivity. However, the release of ions such as calcium and phosphate is essential for this apatite formation process [40].

##### Limitations of SBF testing

SBF testing can yield both false positives and false negatives. For example, glass-ionomers often fail to precipitate apatite in SBF despite showing bioactive properties in vivo, due to factors like the inhibition of calcium phosphate precipitation by poly (acrylic acid) [40].

SBF lacks organic components present in real biological fluids, which can affect its predictive accuracy for in vivo bioactivity [40, 42].

The technique described for ion release analysis using simulated body fluid (SBF) involves two approaches: static and dynamic immersion testing.

##### Approaches of SBF testing

**Static approach:**

Disk-shaped specimens are immersed in a fixed volume of SBF under controlled temperature conditions, allowing for the evaluation of ion release properties by analyzing the SBF at predetermined time intervals to determine the concentration of ions released from the specimens. While this method is straightforward and useful for initial screening, it may not accurately replicate physiological conditions due to the lack of fluid movement, which can lead to saturation effects [43].

**Dynamic approach:**

**Fig. 1:**
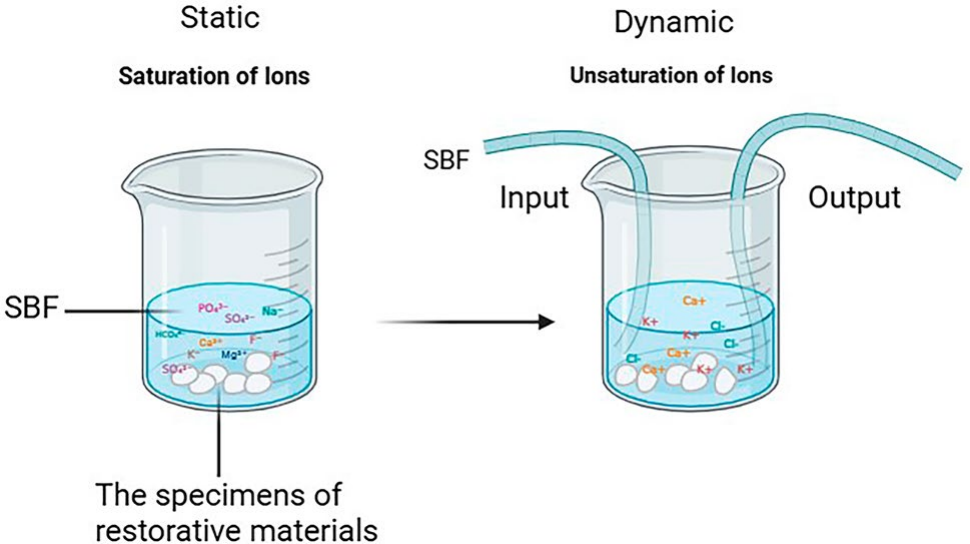
The static and dynamic SBF assay

Simulates a more realistic in vitro environment by allowing a continuous flow of SBF over the specimens. disk-shaped specimens are placed in a specially designed chamber that facilitates fluid flow. The SBF is circulated at a controlled rate and temperature, ensuring fresh fluid continuously interacts with the specimen’s surface Fig. 1. This simulates a more realistic in vitro environment by allowing continuous flow of SBF over the specimens. disk-shaped specimens are placed in a specially designed chamber that facilitates fluid flow. The SBF is circulated at a controlled rate and temperature, ensuring fresh fluid continuously interacts with the specimen’s surface Fig. 1. This setup prevents saturation of released ions in the medium and provides insights into the materials’ ion release behavior under conditions that approximate the dynamic nature of bodily fluids. While dynamic immersion testing is more complex and requires precise control overflow rates and temperature, it offers a more accurate representation of how materials behave in vivo. Together, these techniques are essential for evaluating the biocompatibility and effectiveness of materials intended for medical or dental applications [43, 44]. Comparisons between the two approaches is illustrated in Table 1.

**Table 1:** Comparisons between Static SBF and Dynamic SBF

Static SBF	Dynamic SBF
- Disk specimen in vial	- Specimen in flow chamber
- Stagnant fluid	- Circulating SBF
- Weekly renewal	- Continuous flow (120 rpm)
Outcome: Saturation artifacts	Outcome: Clinically predictive ion release

SBF remains a valuable tool for predicting in vivo bioactivity, particularly when combined with other testing methods. The development of more sophisticated in vitro models that better mimic the oral environment is essential for improving the accuracy of bioactivity assessments.

#### In vivo studies

In addition to in vitro assessments, in vivo studies are conducted to evaluate the apatite-forming ability of hydroxyapatite in a biological environment. For example, new-generation hydraulic calcium silicate cements have been implanted in animal models to observe hydroxyapatite formation over time [45, 46].

#### Method of characterization

In both methods, Changes in morphology and the formation of an apatite layer can be monitored using many characterization techniques. Scanning Electron Microscopy (SEM) is utilized to visualize the surface morphology of hydroxyapatite samples before and after immersion in SBF. This technique allows for observation of apatite layer formation over time, indicating the material’s bioactivity [47]. X-Ray Diffraction (XRD) provides information about the crystallinity and phase composition of synthesized hydroxyapatite. The distinct diffraction patterns help confirm the presence of HAp and assess its purity [47]. Other characterization techniques, such as FTIR spectroscopy and Raman spectroscopy, can provide complementary information about the chemical composition and structure of the apatite layer [45, 46].

Other characterization techniques, such as FTIR spectroscopy and Raman spectroscopy, can provide complementary information about the chemical composition and structure of the apatite layer.

**Fig. 2:**
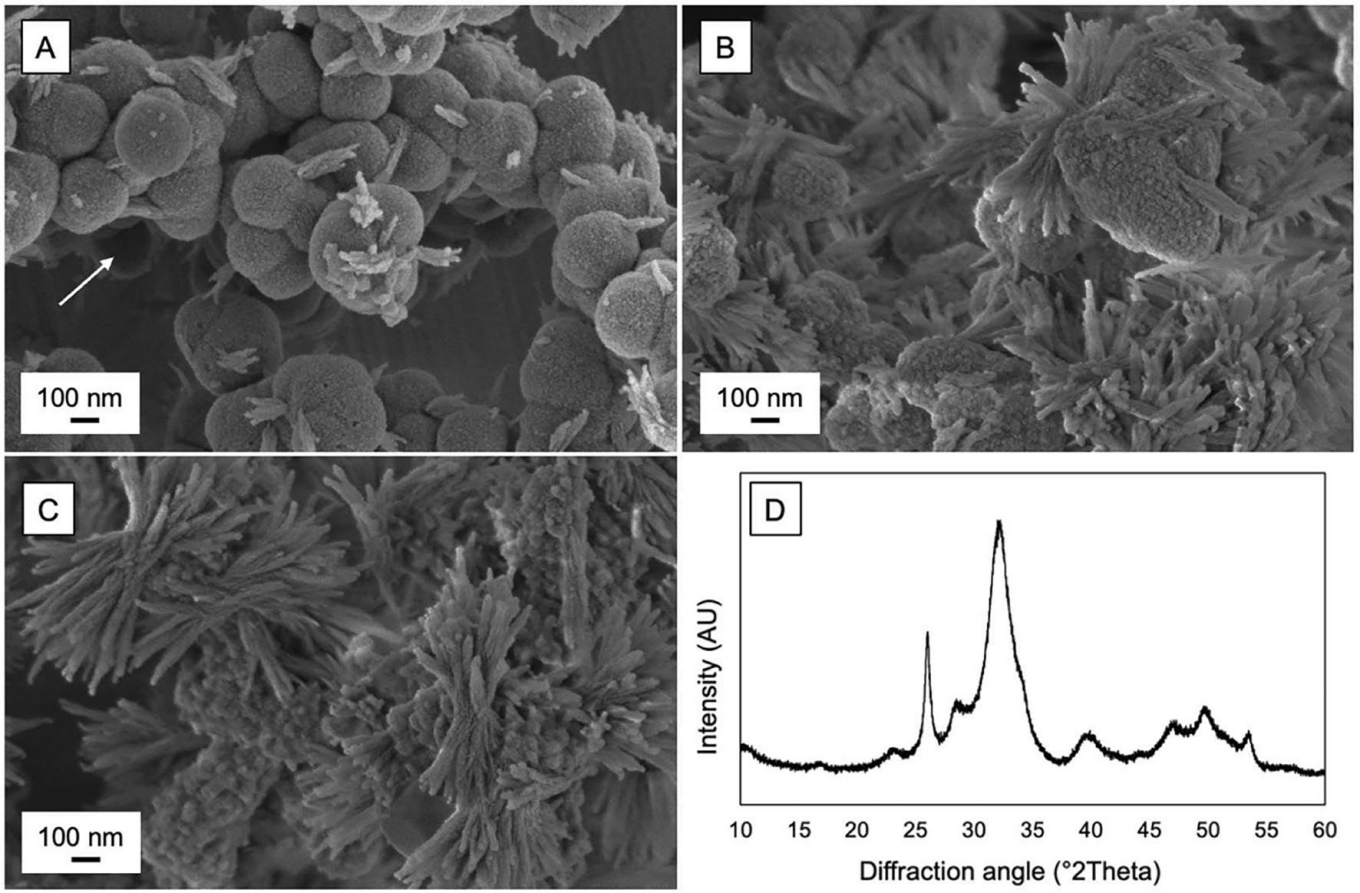
SEM and XRD characterization of amorphous calcium magnesium fluoride phosphate (ACMFP) particles undergoing in vitro crystallization in simulated saliva [48]

Figure 2 shows SEM and XRD characterization of amorphous calcium magnesium fluoride phosphate (ACMFP) particles undergoing in vitro crystallization in simulated sali [48]. (A–C) Sequential SEM images illustrate the progressive transformation from hollow, amorphous core–shell spheres to a dense network of elongated crystalline bundles on the particle surfaces. The arrow in (A) indicates a hollow interior characteristic of the initially amorphous particle core. Over time, high-aspect-ratio crystals emerge and increasingly dominate the particle surfaces (B, C). (D) XRD pattern confirms the conversion of ACMFP into a mixture of hydroxyapatite and fluoride-substituted apatite, revealing the material’s capacity for rapid mineralization under physiologically relevant conditions. Collectively, these findings demonstrate that ACMFP particles release fluoride, calcium, and phosphate ions that drive their own transformation into a crystalline apatite phase. This ability to form apatite-like minerals in a simulated oral environment underpins the particles’ potential for remineralization and acid-resistant repair in dental applications [48].

**Fig. 3:**
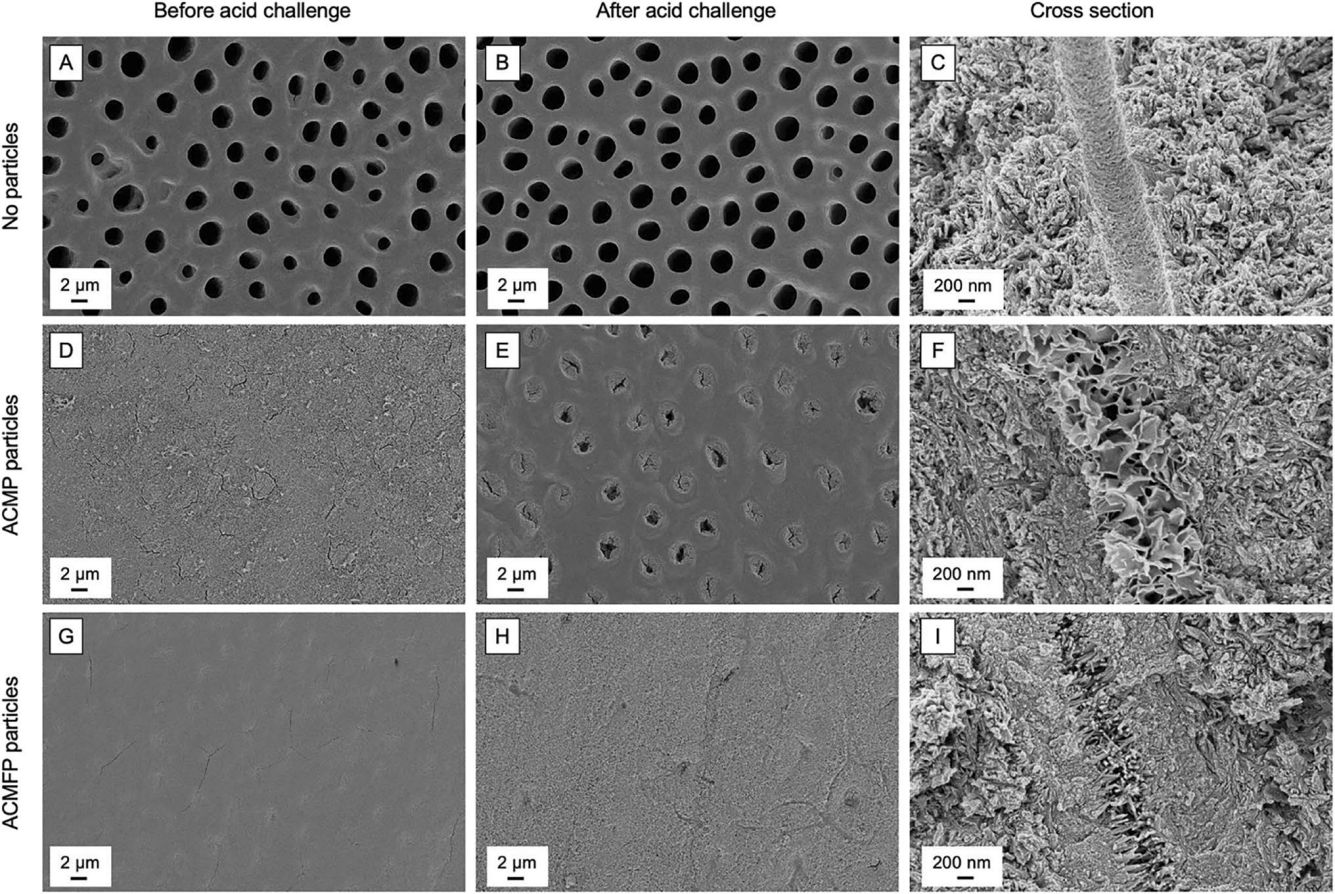
Shows SEM images of dentinal surfaces and dentinal tubules before and after an acid challenge [48]

Figure 3 shows SEM images of dentin surfaces and dentinal tubules before and after an acid challenge, highlighting the impact of adding amorphous calcium phosphate (ACMP) or amorphous calcium magnesium fluoride phosphate (ACMFP) particles on remineralization and tubule occlusion. (A–C) Dentin treated with a blank formula (no mineralizing particles) [48]. The surface (A, B) shows open tubule orifices both before and after acid exposure, illustrating negligible mineral deposition. The cross-sectional view (C) likewise reveals unoccluded tubules. (D–F) Dentin treated with ACMP (no fluoride). Although the tubules are initially filled with a mineralized layer (D), partial reopening is evident post-acid challenge (E). Cross-sectional imaging (F) confirms mineral deposition within tubules, but it is susceptible to acidic dissolution. (G–I) Dentin treated with ACMFP (with fluoride). A continuous, dense mineral layer covers the surface and effectively blocks tubule orifices (G). Even after an acid challenge (H), the tubules remain occluded. Cross-sectional analysis (I) shows a tightly packed mineral front, including high-aspect-ratio crystals extending deep into the tubules. These observations underscore the superior acid resistance conferred by incorporating fluoride into the amorphous calcium phosphate matrix. The ACMFP-treated surfaces and tubules retain their mineralized barrier even under acidic conditions, highlighting the promise of fluoride-bearing amorphous phosphate particles for durable dentin tubule occlusion and enhanced remineralization in clinical dentistry [48].

### Ion release analysis

The bioactivity of restorative materials is primarily linked to their capacity to release fluoride, calcium, and phosphate ions, crucial for promoting remineralization and enhancing antibacterial properties. These ions play a critical role in promoting remineralization and enhancing the material’s antibacterial properties. Fluoride (F⁻) Provides anti-cariogenic effects by inhibiting bacterial metabolism and promoting enamel remineralization. Calcium (Ca^2^⁺) and Phosphate (PO₄^3^⁻) are essential for the formation of hydroxyapatite, contributing to the repair of demineralized tooth structures [49].

The resulting ionic burst elevates the degree of supersaturation, so an amorphous calcium-phosphate (ACP) precursor nucleates on the material surface. *Bakry *et al*.* showed that this transient brushite layer transforms within 24 h into a hydroxyapatite coating enriched with silica, which infiltrates demineralized enamel and restores its micro-hardness [11]. Consequently, the kinetics of ion release and subsequent mineral precipitation are pivotal determinants of the long-term remineralization performance of these materials.

Ion release analysis involves quantifying bioactive ions—namely calcium (Ca^2^⁺), phosphate (PO₄^3^⁻), and fluoride (F⁻)—that are crucial for both remineralization and antibacterial actions. Researchers have experimented with incorporating calcium and phosphate-based compounds into dental materials to encourage the discharge of Ca, P, and F ions [50]. The boost in bioactivity observed in glass ionomer cements (GICs) due to this ion release is key to fostering long-term oral health [50]. For instance, the liberation of Ca and P ions from GIC markedly enhances its bioactive properties [50]. Additionally, the release of these ions can initiate remineralization and help inhibit bacterial growth, thereby supporting overall tooth health [50].

For instance, the liberation of Ca and P ions from GIC markedly enhances its bioactive properties [51]. Additionally, the release of these ions can initiate remineralization and help inhibit bacterial growth, thereby supporting overall tooth health [51]. Many ion release analysis methods used in previous studies are listed in Table 2.

**Table 2:** Show studies measured ion release and measured it with different methods

Study	Material	Methodology	Findings
[52]	Reinforced glass ionomer cement (ChemFil Rock, Dentsply Sirona, Konstanz, Germany; shade: A2, LOT: 1,903,000,819)	1. Atomic absorption spectrometry for calcium 2. UV–Vis spectroscopy for phosphate 3. Ion-selective electrode for fluoride	1. Resin-based adhesive layers may impede ion release, thereby limiting the advantages offered by remineralizing restorative materials 2. Sufficient water uptake is necessary to promote ion release from filler components
[53]	Glass-ionomer cement (AquaCem, Dentsply, Konstanz, Germany)	1. Atomic absorption spectrometry for calcium 2. Ion-selective electrode for fluoride (Phosphate ion release was not examined)	1. Incorporation of chicken eggshell powder: – Improves mechanical characteristics – Exerts no noteworthy impact on fluoride and calcium release
[54]	Glass ionomer cement (Fuji VIITM [F7] and Fuji VIITM EP [F7EP])	1. Atomic absorption spectrometry for calcium 2. UV–Vis spectroscopy for phosphate 3. Ion-selective electrode for fluoride	1. Adding 3% (w/w) CPP–ACP to the GIC: – Elevates calcium and phosphate ion output – Does not significantly change fluoride release, surface hardness, or mass
[55]	Glass ionomer cement (AquaCem, Dentsply)	1. Inductively coupled plasma-optical emission spectroscopy for calcium 2. Inductively coupled plasma-optical emission spectroscopy for phosphate (Fluoride release was not investigated)	1. Calcium ions remain unreleased under neutral conditions but are notably released in acidic environments 2. Phosphate ions are released under both neutral and acidic conditions, with higher release occurring in acidic settings
[56]	Conventional aluminosilicate glass (fabricated by the researcher)	1. Atomic absorption spectrometry for calcium 2. UV–Vis spectroscopy for phosphate (Fluoride ion release was not investigated)	1. Introducing bioactive glass increases bioactivity through apatite formation but compromises mechanical strength 2. Adding Al^3^⁺ to the bioactive glass boosts strength while reducing bioactivity

#### Techniques for measuring ion release

##### Ion-selective electrode (ISE)

This method utilizes an electrode that selectively responds to fluoride ions in solutions. The potential difference generated by fluoride and Calcium ions is measured against a reference electrode, providing a direct indication of fluoride concentration [57].

##### Atomic absorption spectroscopy (AAS)

This method measures calcium and Phosphate concentrations by analyzing the absorption of light at a specific wavelength characteristic of calcium. Involves reactions that produce a color change proportional to the concentration of the ion, quantified via spectrophotometry [58]. The samples are immersed in a solution (e.g., distilled water) then the washed-out solution after a predetermined time is collected to analyze calcium release. If analyzing a solid sample, it must be dissolved in an acid to free calcium ions. The concentration of calcium in the sample can be determined using the calibration curve. Standard solutions of calcium are prepared at known concentrations for creating a calibration curve.

##### Inductively coupled plasma optical emission spectrometry (ICP-OES)

This analysis is performed using ICP-OES, which identifies and measures the concentrations of specific ions, such as calcium or phosphorus. The specimens are immersed in a liquid medium, such as distilled water or simulated body fluid, which is analyzed periodically to detect and quantify ions released from the specimens. The method is known for its sensitivity and ability to detect multiple elements simultaneously, making it suitable for evaluating the ionic release characteristics of dental materials [46].

### pH variation and neutralizing capacity assessment

The assessment of pH variation and neutralizing capacity is crucial in evaluating the interactions of dental materials with their environment, particularly in the oral cavity. This analysis helps understand how these materials respond to acidic conditions, which can occur due to dietary habits or pathological conditions like gastroesophageal reflux disease (GERD) [59]. GICs typically exhibit an acidic pH during the initial setting phase, which gradually neutralizes over time. This pH change is significant for ion release but may limit the formation of hydroxyapatite under acidic conditions. Despite their ion-releasing capabilities, conventional GICs have limited antibacterial activity and can exhibit cytotoxicity over time. These shortcomings highlight the need for modifications to enhance their bioactive properties [60].

#### pH variation assessment

pH variation assessment typically involves immersing dental materials in different pH media for specified durations, and their pH is measured at regular intervals using a calibrated pH meter. Calibration of the pH meter is performed using standard buffer solutions with known pH values to ensure accurate readings. The immersion conditions, including temperature and solution replacement schedule, are controlled and standardized [46]. This method allows researchers to monitor how the material influences the surrounding pH over time.

#### Neutralizing capacity assessment

Neutralizing capacity refers to the ability of dental materials to buffer or neutralize acidic conditions in their surroundings. This property is particularly important for materials used in restorative dentistry, as they can help mitigate the effects of acid exposure on tooth structure and overall oral health. To assess neutralizing capacity, specimens are typically immersed in an acidic solution (e.g., hydrochloric acid) at a controlled pH [61]. Following immersion, the change in pH of the surrounding solution is measured over time. A material with high neutralizing capacity will demonstrate a significant increase in the solution’s pH as it buffers the acidity. This assessment can be complemented by analyzing ion release (e.g., calcium and fluoride ions) during immersion, as these ions can contribute to the buffering capacity [62].

### Hydroxyapatite formation assessment

#### Remineralization potential

The assessment of remineralization potential employs a variety of methods, including SEM–EDX analysis, Vickers Microhardness testing, CLSM, pH-cycling models, and light scattering techniques. Each method offers unique insights into the effectiveness of different remineralizing agents and their ability to restore lost minerals in dental tissues. Understanding these methodologies is essential for developing effective treatments aimed at enhancing oral health and preventing caries progression.

##### Scanning electron microscopy (SEM) and energy dispersive X-ray (EDX) analysis

**Fig. 4:**
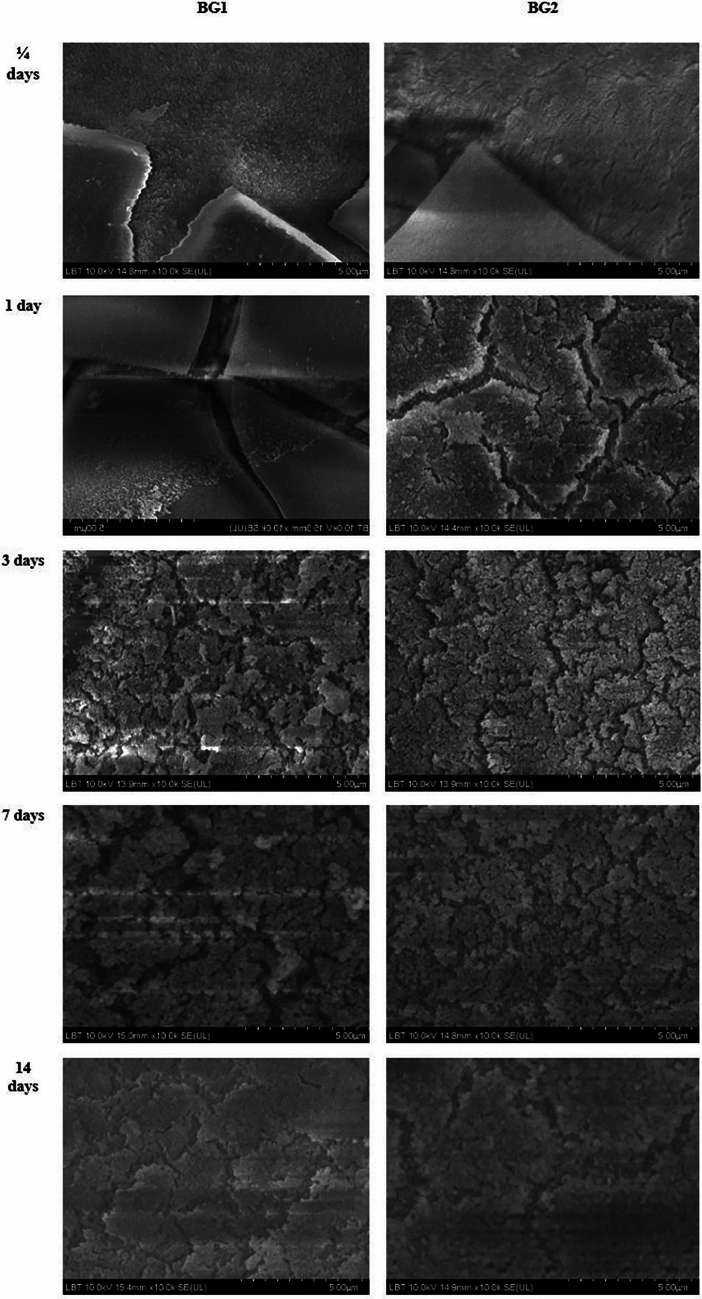
Shows SEM micrographs depicting the surface evolution of the two newly synthesized bioactive glasses—BG1 (left column) and BG2 (right column)—after immersion in PBS at 37 °C for 1/4, 1, 3, 7, and 14 days

SEM is widely used to assess the surface morphology of enamel before and after treatment with remineralizing agents [63, 64]. This technique allows for high-resolution imaging of the enamel surface, revealing structural changes indicative of remineralization. Figure 4 shows SEM micrographs depicting the surface evolution of the two newly synthesized bioactive glasses—BG1 (left column) and BG2 (right column)—after immersion in PBS at 37 °C for 1/4, 1, 3, 7, and 14 days. Initially, smooth surfaces in both materials acquire increasingly irregular deposits, becoming progressively denser and more compact over time. Compared to BG1, BG2 demonstrates a more pronounced texture change at earlier immersion intervals, suggesting faster interaction with the surrounding medium. These morphological shifts are consistent with the gradual formation of a surface layer (likely hydroxyapatite or other Ca/P-rich phases), reflecting the in vitro biodegradability and bioactivity of the glass compositions [65].

EDX, often coupled with SEM, provides elemental analysis by measuring the composition of calcium (Ca) and phosphorus (P) in the enamel. The Ca/P ratio is a critical indicator of remineralization, as it reflects the mineral content of the treated enamel compared to untreated or demineralized samples. For instance, studies have shown that specimens treated with different remineralizing agents exhibit varying degrees of surface smoothness and mineral content when analyzed using SEM–EDX [63]. The mineralization of the bioactive glass with high phosphorus (10.8 mol% P(_2_)O(_5_)-54.2 mol% SiO(_2_)-35 mol% CaO, named PSC) and its ability to induce type I collagen mineralization were observed by SEM and TEM [65].

##### Vickers microhardness test (VMH)

It is another quantitative method used to evaluate the hardness of enamel before and after remineralization treatments. This test measures the resistance of the enamel surface to indentation, providing insights into changes in mineral content. A statistically significant increase in microhardness after treatment indicates effective remineralization [66].

##### Confocal laser scanning microscopy (CLSM)

CLSM is employed to visualize demineralization and remineralization processes at a microscopic level. This technique allows for three-dimensional imaging of tooth structures and can assess areas of mineral loss and gain following treatment with remineralizing agents. In studies utilizing CLSM, samples are often stained with dyes (e.g., rhodamine B) to enhance visualization of demineralized areas, enabling researchers to quantify changes in mineral content over time [63].

##### pH-cycling models

In vitro pH-cycling models simulate the dynamic conditions found in the oral cavity by alternating between demineralizing and remineralizing phases. This method allows researchers to assess how effectively a material can promote remineralization under conditions that mimic real-life exposure to acids from dietary sources. The effectiveness of different remineralizing agents can be compared based on their ability to restore mineral content during these cycles.

##### Light scattering techniques

Light scattering techniques can also be utilized to assess changes in mineral content within enamel samples. These methods measure variations in light transmission through the enamel before and after treatment, providing indirect evidence of remineralization based on changes in optical properties.

Bioactive materials can be used as direct restorative materials, enhancing the bond between the restoration and dental tissues while promoting remineralization [1].

## Clinical applications of bioactive glasses

**Fig. 5:**
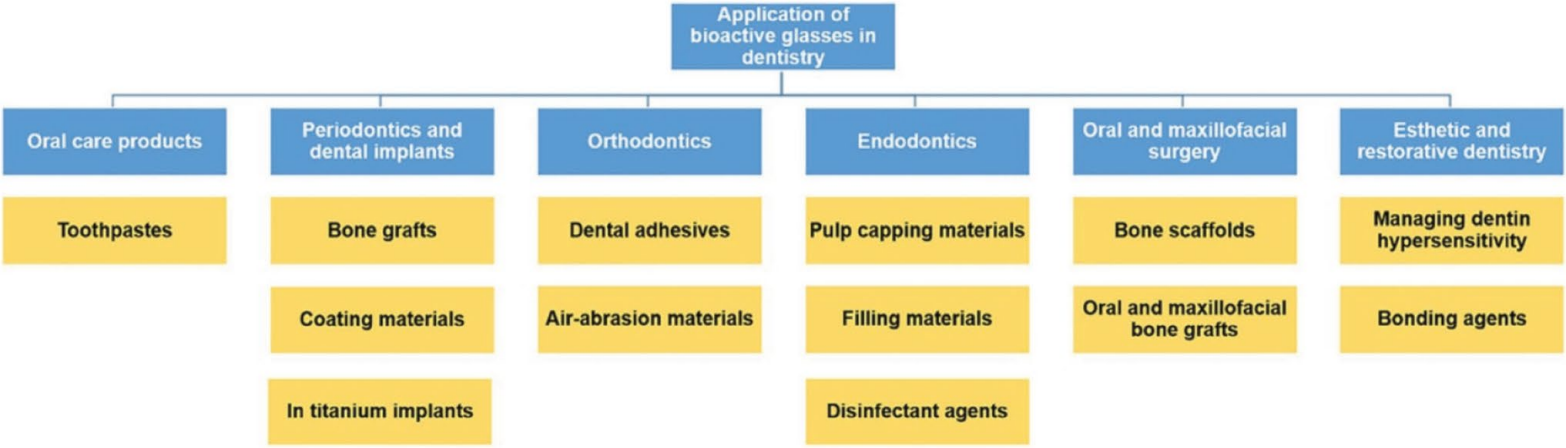
Represents a schematic overview of the diverse dental applications of bioactive glasses

Bioactive glass materials offer broad utility, from oral care products (such as toothpastes that aid in remineralization and sensitivity reduction) through periodontic interventions (bone grafts, implant coatings), orthodontic adhesives, and endodontic treatments (pulp-capping, filling, and disinfection). Their role extends into oral/maxillofacial surgeries (bone scaffolds and grafts) as well as esthetic and restorative dentistry (e.g., dentin hypersensitivity management and bonding agents). Collectively, this diagram underscores the versatility and clinical significance of bioactive glasses in fostering tissue regeneration, enhancing antimicrobial properties, and improving mechanical stability in various dental procedures [67]. Figure 5 represents a schematic overview of the diverse dental applications of bioactive glasses.

This review is concerned with the influence of bioactive materials on direct restorative materials such as glass ionomer cements (GICs), resin-based composites, and ion-releasing materials.

## Bioactivity of glass ionomer cements

Glass ionomer cements can release and replenish fluoride ions, thus potentially aiding remineralization while also forming a direct bond with the tooth’s hard tissue [68, 69]. However, their mechanical performance tends to be inadequate [68]. Leading to higher failure rates and rendering them unsuitable as a direct restoration capable of handling occlusal force. Consequently, improving the bioactivity of GICs remains a critical goal for enhancing their clinical efficacy. One approach involves incorporating bioceramic particles—such as bioactive glass, calcium phosphate, and amorphous calcium phosphate—into dental materials to help prevent caries and support tooth remineralization [70]. Nonetheless, the ion release from these additives can generate voids, potentially undermining both the physical and mechanical properties of the materials [71].

### The interaction of glass-ionomers with biological tissues

In view of the recent policy statement by the FDI (World Dental Federation) on the bioactivity of dental restorative materials, several overarching points emerge regarding the interaction of glass-ionomers with biological tissues [10].

#### Ion release

Both variants of glass polyalkenoate cement have been shown to emit various ions under neutral conditions—such as sodium, aluminium, silicate, phosphate, and fluoride—and either calcium or strontium in acidic conditions [55].

#### pH modification

These cements alter the pH of the surrounding environment, a phenomenon initially described as buffering, attributed to the partial neutralization of a weak acid within the cement [72].

#### Robust bonding

They establish a mechanically strong bond with both enamel and dentine [73].

#### Demonstrated bioactivity

They exhibit bioactivity under in vitro conditions [74].

### Evidence of bioactive behavior of GICs

GICs do not always show apatite precipitation in simulated body fluid (SBF) GICs have been shown to bond effectively to living bone and hard tissues in vivo, indicating bioactive properties. The presence of poly (acrylic acid) (PAA) in GICs can inhibit apatite formation in SBF, leading to false negatives in bioactivity tests. Even small amounts of PAA can significantly reduce apatite precipitation [75]. While GICs may not always meet traditional criteria for bioactivity based on SBF testing, they exhibit bioactive properties through ion release, mechanical bonding, and positive cell interactions. These characteristics support their classification as bioactive materials in the context of restorative dentistry.

The interaction of 45S5 Bioglass and biological environments, such as simulated body fluid (SBF), is crucial for understanding how these materials can induce apatite formation and integrate with tissues like bone and dentine. Here are key points related to bioactive glass and its effects:

#### Apatite formation mechanism

5S5 Bioglass, a well-known type of bioactive glass, promotes apatite formation through a series of ion exchange reactions when immersed in SBF. This process involves of calcium and phosphate ions, which eventually precipitate as hydroxyapatite, a mineral similar to bone tissue [76, 77].

##### Role of bioactive glass powder

Adding 45S5 Bioglass powder to materials like glass polyalkenoates enhances the release of calcium, silica, and phosphate ions, which is essential for apatite deposition from SBF [76].

##### Effect without bioactive glass

Without bioactive glass, conventional or resin-modified glass polyalkenoates do not induce apatite precipitation in SBF. However, they still exhibit beneficial effects in vivo by forming strong bonds with hard tissues due to their ion-release properties [76].

##### In vivo and in vitro effects

Despite the inability to induce apatite formation in vitro, glass polyalkenoates show positive effects in vivo. They form strong mechanical bonds with dentine, enamel, and bone, which is attributed to their ion-release capabilities [40]. In cell culture studies, resin-modified glass-ionomers, even without bioactive glass powder, support cell attachment and proliferation. This suggests that these materials can interact positively with biological systems at the cellular level [40].

#### Bioactive glass composition and properties

Bioactive glasses can be modified with various elements such as strontium (Sr), zinc (Zn), and copper (Cu) to enhance their bioactivity and control their degradation rates. For example, Sr incorporation increases the rate of hydroxycarbonated apatite deposition [78].

The reactivity of bioactive glass is significantly influenced by its surface area. Glasses with higher surface areas, such as those produced via the sol–gel method, exhibit faster ion exchange and apatite formation rates [40].

### Modifications to enhance bioactivity

Over the years, various modifications have been explored to enhance their mechanical properties, bioactivity, and biological responses, making them more effective for diverse clinical applications. This table summarizes recent studies on bioactive GICs, focusing on materials studied, research aims, bioactivity evaluations, outcomes, and key findings. These studies highlight advancements in incorporating bioactive additives, nanoparticles, and novel composites into GIC formulations, aiming to improve ion release, remineralization potential, antibacterial properties, cytotoxicity, and mechanical strength. This overview provides valuable insights into the evolving field of bioactive GICs and their potential to address current limitations and broaden their scope in restorative dentistry. Recent modifications un GIC regarding bioactivity ae shown in Table 3.

**Table 3:** Recent modifications un GIC regarding bioactivity

Study	Materials studied	Aim	Bioactivity evaluation	Out come	Key findings	Publication
Are glass ionomer cements bioactive? Analysis of ions release, pH, hydroxyapatite nanoprecursors formation, antibacterial activity, and cytotoxicity	Six commercial GICs (e.g., Bioglass, Ionglass)	Six commercial glass ionomer cements (GICs) were analyzed for the release of F⁻, Ca^2^⁺, and PO₄^3^⁻ ions, their alkalinizing effects, cytotoxicity, antibacterial properties, and their capacity to form hydroxyapatite nanoprecursors	Ion release, pH analysis, SEM/EDS, FTIR, XRD, cytotoxicity, antibacterial activity	Bioactivity	GICs released ions for remineralization but lacked antibacterial activity, hydroxyapatite precursors, and showed cytotoxicity after 72 h	[79]
Fabrication and characterization of reinforced glass ionomer cement by zinc oxide and hydroxyapatite nanoparticles	By zinc oxide and hydroxyapatite nanoparticles	Enhancement of glass ionomer cement (GIC) by incorporating hydroxyapatite (HA) and zinc oxide (ZnO) nanoparticles to improve its mechanical strength, biological activity, and antibacterial properties	Scanning Electron Microscopy (SEM) was employed to monitor the development and growth of calcium formations pH Fluoride release evaluation Antibacterial examination Cytotoxicity	Bioactivity Cytotoxicity compressive strength	Adding HA and ZnO substantially enhanced the bioactivity, mechanical strength, and antibacterial performance of the glass cement. Notably, incorporating 4 wt% ZnO achieved the highest improvement in compressive strength while maintaining a favorable cytocompatibility profile. However, this modification also led to a reduction in fluoride release, indicating a trade-off between mechanical improvements and fluoride ion availability	[80]
Soda-lime-silica glass/tetracalcium phosphate	Modified GIC with SLS/TTCP composite	The study investigates how the inclusion of soda-lime-silica glass (SLS) and tetracalcium phosphate (TTCP) composites (SP) affects the mechanical and physical properties of conventional glass ionomer cement (GI) restorations	The formation of the hydroxyapatite (HA) layer was examined using FTIR, SEM–EDS, and XRD techniques The release of ions, specifically phosphorus and calcium ions, along with pH variations, was quantified using inductively coupled plasma-atomic emission spectroscopy (ICP-AES) The cytotoxic effects on normal human fibroblasts were evaluated Antimicrobial activity	Setting time, Bioactivity, microhardness, cytotoxicity, antibacterial activity	Enhanced ion release (Ca^2^⁺, PO₄^3^⁻), improved microhardness, and reduced cytotoxicity; effective in inhibiting bacterial growth	[81]
Acemannan-containing bioactive resin modified glass ionomer demonstrates satisfactory physical and biological properties	Acemannan is recognized as the predominant polysaccharide derived from Aloe vera	Develop acemannan-containing bioactive resin-modified glass ionomers (RMGIs)	Fluoride release. Cytotoxicity and growth factors secretion	Evaluations include the depth of cure, flexural strength, and fluoride release, along with assessments of cytotoxicity and the secretion of growth factors	Incorporating acemannan into resin-modified glass ionomer cements (RMGIs) reduces certain mechanical properties while enhancing some biological activities. This outcome suggests that RMGIs enriched with acemannan could serve as effective lining materials for dentin-pulp regeneration	[82]
The development of resin-coating materials for enhancing elemental release of coated glass ionomer cements	The resin coating is supplemented with additives such as monocalcium phosphate monohydrate (MCPM), Sr/F-doped bioactive glass (Sr/F-BAGs), and pre-reacted glass ionomer fillers (SPG)	The study compares the degree of monomer conversion (DC), biaxial flexural strength, surface microhardness, and ion release of glass ionomer cements coated with experimental materials to those coated with a commercial product (EQUIA Coat, EC)	The release of fluoride along with other elements (Ca, P, Sr, Si, Al) is quantified using fluoride-specific electrodes and inductively coupled plasma-optical emission spectroscopy (ICP-OES)	The analysis focuses on the degree of monomer conversion (DC), biaxial flexural strength, surface microhardness, and the overall bioactivity of the material	Glass ionomer cements treated with the experimental resin coating containing ion-releasing additives showed mechanical properties comparable to the commercial standard, while also achieving higher ion release. This enhanced ion release could potentially improve the remineralization effects of the coated GICs	[83]
Bioactive Self-Polymerizing Resin with Surface Pre-Reacted Glass Ionomer Fillers for Suppressed Enamel Demineralization	Surface Pre-Reacted Glass Ionomer Fillers (S-PRG)	The aim is to formulate a bioactive auto-polymerizing resin (APR) by integrating surface-pre-reacted glass ionomer (S-PRG) fillers in different amounts and to evaluate its bioactivity, focusing on acid buffering, ion release, and its ability to inhibit enamel demineralization	pH neutralization, ion release, and inhibition of enamel demineralization	Bioactivity	The newly developed auto-polymerizing resin containing S-PRG filler effectively releases ions and maintains a low-pH solution within a neutral range, thereby preventing enamel demineralization. This demonstrates that S-PRG fillers can be effectively utilized in prosthetic applications as a bioactive material to protect against enamel demineralization	[84]
Evaluation of compressive and diametral tensile strength of novel bioactive material with conventional glass ionomer cement and silver amalgam: An in vitro study	Zirconia-reinforced GIC		–	Compressive strength, diametral tensile strength	Silver amalgam has the highest compressive and DTS among all the direct restoratives employed. Zirconomer showed compressive and DTS greater than that of the unmodified GIC, such as Fuji IX and Shofu FX II	
Bioactive Compounds Enhance the Biocompatibility and the Physical Properties of a Glass Ionomer Cement	The flavonoids apigenin, naringenin, quercetin, and liquiritigenin	To evaluate the toxicological effects and physical properties of glass ionomer cement (GIC) modified with flavonoids (apigenin, naringenin, quercetin, and liquiritigenin) to characterize the impact of these bioactive compounds on the material’s performance and biocompatibility	–	Cytoxicity, compressive strength, surface roughness, and hardness	Increased the biocompatibility of GIC to keratinocyte cells. Their surface hardness was enhanced without modifying other physical properties	[85]
Effects of novel additives on the mechanical and Biological properties of glass ionomer cement: An in vitro study	Hydroxyapatite, multi-walled carbon nanotubes, graphene, and bioactive glass	The aim was to determine if integrating innovative additives into Glass Ionomer Cement can enhance its biocompatibility and mechanical attributes	–	The investigation measured compressive strength, microhardness, and diametral tensile strength, in addition to assessing in vitro cytotoxicity as an indicator of biocompatibility	The GIC formulation enhanced with graphene demonstrated superior physical properties—exhibiting higher compressive strength, diametral tensile strength, and microhardness—when compared to the other formulations. Furthermore, regarding biocompatibility, the HAP, Graphene, BAG, and conventional GIC groups did not exhibit cytotoxic effects, while the group containing multiwalled carbon nanotubes showed signs of cytotoxicity	[86]
Comparison of remineralization ability of tricalcium silicate and of glass ionomer cement on residual dentin: an in vitro study	Biodentine	Compare the remineralization effects of a calcium silicate-based cement (Biodentine) and of a glass ionomer cement (GIC: Fuji IX) on artificially demineralized dentin	Remieneralization potential	Remieneralization potential (SEM–EDX)	The dentin lesion remineralization capability of Biodentine is higher than that of GIC, suggesting the usefulness of the former as a bioactive dentin replacement material	[87]
Developing a novel glass ionomer cement with enhanced mechanical and chemical properties	Nanosilver doped bioactive glass (NanoAg BAG)	Create an innovative glass ionomer cement (NGIC) that boasts superior mechanical and chemical attributes, and evaluate its biocompatibility, structural strength, and ion release performance	An analysis was conducted on the NGIC to investigate its biocompatibility, surface features, elemental makeup, topographical details, chemical characteristics, compressive and diametral tensile strength, as well as its ion release profile		A NGIC exhibiting both improved biocompatibility and mechanical performance was formulated, with a 10% incorporation of PVPA proving to deliver the most significant enhancements in these areas	[88]
Enhanced remineralisation ability and antibacterial properties of sol–gel glass ionomer cement modified by fluoride containing strontium-based bioactive glass or strontium-containing fluorapatite	Fluorinated bioactive glass (SrBGF) or strontium-containing fluorapatite (SrFA)	The study compared two distinct bioactive additives—strontium-enriched fluorinated bioactive glass (SrBGF) versus strontium-containing fluorapatite (SrFA)—incorporated into sol–gel derived glass ionomer cement (SGIC)	Remineralisation, ion-releasing	Antibacterial activity, remineralisation, and hASCs and hDPSCs viability. Surface roughness and ion-releasing behavior	SrFA exhibited superior antibacterial properties compared to SrBGF while maintaining an equivalent ability to induce apatite crystal precipitation and promote remineralization. SGIC modified with SrBGF or SrFA showed promising effects on the in vitro cytotoxicity of hASC and hDPSC	[89]

## Dental composite resin

Dental composite resin is an interesting area of research work. It has been modified by many additives to enhance its properties [90]. Various calcium phosphates—including monocalcium phosphate, dicalcium phosphate, tricalcium phosphate, hydroxyapatite (HA), and amorphous calcium phosphate (ACP) are utilized as ion-releasing fillers in experimental composite materials. Among these, ACP is particularly noteworthy because it directly precedes the formation of HA, a key component in the natural mineralization process of teeth and bones. Typically, the performance of ACP-based composites is evaluated by measuring the concentration of released calcium and phosphate ions in an aqueous solution. As a result, composites containing ACP that generate calcium/phosphate solutions supersaturated with HA are considered capable of remineralizing dental hard tissues [91].

Beyond purely chemical evidence of their demineralizing potential, the bioactive properties of ACP composites have been confirmed through in vitro [92], in situ [93] and in vivo [94].

Bioactive glasses (BGs), such as 45S5 Bioglass and Biomin, are soluble materials primarily formulated from SiO₂, CaO, Na₂O, and P₂O₅ in various proportions [95]. Their solubility and bioactivity are directly influenced by their specific compositions, which allows them to be tailored for particular applications [95]. Historically, BGs have been employed in orthopedic settings, notably as coatings for bone implants, because of their ability to precipitate hydroxyapatite (HA) and thereby enhance osseointegration. [26]. In experimental dental composites, BGs hold potential as sources of remineralizing ions. Upon exposure to water, BGs undergo a series of reactions, including ion exchange, dissolution, and reprecipitation, leading to HA formation [95].

To enable these effects, the composite must allow water access to BG particles, ensure ion release, and avoid silane-coated BG particles. Additionally, resin hydrophilicity must be optimized [96].

### Modifications of dental composite resin

Modifications of GIC have been a focus area of research recently. Recent modifications are listed in Table 4.

**Table 4:** Modification of dental composite resin

Title	Aim	Additive	Significance	Publication
Urchin-like multiscale structured fluorinated hydroxyapatite as versatile filler for caries restoration dental resin composites	In this study, inspired by plant roots’ ability to stabilize and improve soil, fluorinated urchin-like hydroxyapatite (FUHA) with a three-dimensional whisker structure and bioactive components of calcium, phosphorus, and fluorine was designed and synthesized by a dynamic self-assembly method	urchin-like hydroxyapatite (FUHA	FUHA with 50 wt% loading in resin matrix endowed DRC (F5) with excellent physicochemical properties, dentin remineralization property, cell viability, promotion of dental pulp stem cells mineralization, and antibacterial properties. Meanwhile, F5 also presented good clinical handling and aesthetic characteristics	[97]
Niobium oxyhydroxide as a bioactive agent and reinforcement to a high viscosity bulk-fill resin composite		Niobium oxyhydroxide	: The high-viscosity bulk-fill resin composite with 0.5% niobium oxyhydroxide fillers showed promising outcomes as reinforcement agents and performed well for bioactive potential, although less predictable than the commercial resin composite with Giomer technology (Beautifil Bulk U)	[98]
Investigation of mechanical properties, remineralization, antibacterial effect, and cellular toxicity of composite orthodontic adhesive combined with silver-containing nanostructured bioactive glass	The aim of this study was to produce a composite orthodontic adhesive combined with nano-bioactive glass-silver (nBG@Ag) for bracket bonding to enamel and to investigate its cytotoxicity, antimicrobial activity, remineralization capability, and bond strength	Nano-bioactive glass-silver (nBG@Ag)	It demonstrates high biocompatibility while maintaining mechanical properties, particularly shear bond strength. This innovative composition promotes the formation of a hydroxyapatite layer on enamel surfaces, resulting in enhanced antimicrobial properties and reduced enamel demineralization	[66]
Marginal integrity of prototype bioactive glass-doped resin composites in class II cavities	This in vitro study examined the marginal integrity of experimental composite materials doped with bioactive glass (BG)	BG 45S5 (C-20), fluoride-containing BG (F-20),	experimental composites with BG showed at least as good marginal adaptation as the commercial reference, with an indication of possible re-sealing of marginal gaps	[99]
Retention of strength and ion release of some restorative materials	This study aimed to investigate the retention of strength in accelerated aging condition and ion release from an experimental fiber-reinforced bioactive flowable composite resin (Bio-SFRC), comparing it with various commercially available ion-releasing materials	borosilicate glasses containing TiO₂ and ZnO, carbonated apatite, and calcium carbonate particles	Bio-SFRC, an experimental light-cured fber-reinforced flowable composite, had higher flexural strength values before and after hydrothermal aging compared to several commercial ion-releasing materials. The advantages of its fiber-containing structure and slow release of ions suggest that Bio-SFRC is a promising bioactive material to provide ions for mineralization of surrounding tissues, and keeping the durability of the materials at a higher level than that of other tested materials	[44]
The influence of copper-doped mesoporous bioactive nanospheres on the temperature rise during polymerization, polymer cross-linking density, monomer release and embryotoxicity of dental composites	Composites with copper-doped mesoporous bioactive nanospheres (Cu-MBGN) were developed to prevent secondary caries by imparting antimicrobial and ion-releasing/remineralizing properties	Cu-MBGN	The composite with 5 wt% Cu-MBGN combined with nanosilica fillers showed the lowest ethanol softening, indicating the polymer’s highest durability and cross-linking density. Despite the TEGDMA released from all tested materials, no embryotoxic effect was observed	[100]
Physical and mechanical characterizations of experimental pit and fissure sealants based on bioactive glasses	To synthesize and characterize experimental pit and fissure sealants, comparing their properties with those of a commercially available sealant	Biomin F, Biomin C, and S53P4	The study demonstrated that experimental pit and fissure sealants incorporating bioactive glasses, specifically Biomin F powder, offer promising improvements in certain surface properties, such as reduced surface roughness and a lower water contact angle, compared to a commercially available sealant (Seal-Rite). While the mechanical properties, such as nanohardness and elastic modulus, were generally higher in the control group, G1 (Biomin F powder) showed no significant difference from the control group, indicating its potential as a viable alternative	[101]
POSS hybrid bioactive glass dental composite resin materials: Synthesis and analysis	Create a dental composite by hybirding polyhedral oligo-sesquioxide nano monomers and bioactive glass BG 45S5	BG 45S5	The hybrid composite resin containing 20% bioactive glass and up to 4% POSS demonstrated excellent mechanical properties, ion release capability, and bioactivity without compromising biocompatibility	[102]

## Ion releasing direct restorative materials

Direct restorative materials that release therapeutic ions, such as fluoride and calcium, represent a great shift in dentistry. These materials aim to bridge the gap between traditional composites and glass ionomer cements (GICs), offering both mechanical strength and bioactivity. Among the recent advancements, Two direct restorative materials that release ions have reached the market: Cention N (CN) from Ivoclar Vivadent in Schaan, Liechtenstein, and ACTIVA™ Bioactive Restorative (ACT) from Pulpdent in Massachusetts, USA.

### ACTIVA™ bioactive restorative (ACT)

The manufacturer markets ACT as a “bioactive composite” that allegedly releases more fluoride than traditional glass ionomer cements (GICs) [103], though this claim has been challenged [104]. Some researchers even view ACT as an enhanced, reinforced version of a resin-modified glass ionomer (RMGIC) because it incorporates a modified polyacid containing a small amount of water and follows similar chemical setting reactions [105]. Additionally, ACT is formulated with a proprietary bioactive matrix and specialized bioactive fillers [104]. It is promoted as a highly esthetic composite that not only embodies the benefits of glass ionomers—such as chemically bonding to teeth and sealing against microleakage—but also provides a robust, resilient resin matrix that resists chipping and crumbling. Moreover, ACT is reported to release higher levels of calcium, phosphate, and fluoride, making it more bioactive than conventional glass ionomers and more durable and fracture-resistant than standard composites. Its design enables a continuous cycle of ion release and recharge—vital minerals naturally present in saliva and supported by dietary sources—as this dynamic ion diffusion can only occur in materials capable of water transport, a property absent in traditional hydrophobic materials.

### Cention N (CN)

In contrast, CN is described by its manufacturer as an “alkasite”—a variant of resin-based composites that functions as a self-adhesive composite. It contains three primary fillers: inert barium alumino-silicate glass, calcium barium alumino-fluoro-silicate glass, and an active fraction of calcium fluoro-silicate glass [106].

Comparative evaluations have shown that both ACT and CN release varying amounts of ions, including fluoride (F⁺) and calcium (Ca^2^⁺) [107]. However, there is not yet sufficient evidence to confirm any bioactivity for either material. For example, Garoushi et al. noted no mineralization potential for ACT despite its moderate calcium release [108]. Furthermore, Tiskaya et al*.* [109] found that CN released higher levels of F⁺ and Ca^2^⁺ and was capable of forming an “apatite-like” phase. Although these findings suggest a potential bioactive advantage for CN, additional studies are necessary for confirmation. Both materials are considered bulk-fill restorative options. The depth of cure (DoC) is crucial since inadequate curing can lead to problems such as gap formation, marginal leakage, recurrent caries, adverse pulpal effects, and ultimately restoration failure [110]. Given that ion-releasing materials are inherently brittle and prone to cracking, measuring fracture toughness (KIC) is an appropriate method to assess their strength [111]. This property is also linked to the clinical fracturing of resin composites, making it a key parameter for the long-term success of direct restorations [112]. Moreover, evaluating durability through degradation methods like water immersion is essential [113]. As is assessing wear resistance, particularly in large restorations or in patients with bruxism or clenching habits [70].

Notably, after one day of water storage, ACT exhibited the highest fracture toughness; however, after 30 days, both ACT and CN performed similarly to conventional resin-based composites (RBCs) [114]. Water storage significantly diminished the fracture toughness of both ACT and CN, suggesting potential instability and a risk of hydrolytic degradation, and both materials also experienced markedly greater wear compared to conventional RBCs or RMGICs [114].comparison between CN and ACT is summarized in (Table 5).

**Table 5:** Comparison between Cention N (CN) and ACTIVA™ Bioactive Restorative (ACT)

Property	Cention N (CN)	ACTIVA™ Bioactive Restorative (ACT)
Category	Alkasite (Resin-based composite)	Bioactive composite (Resin-modified glass ionomer-like)
Setting Mechanism	Light-curing and optional self-curing	Dual cure (RMGIC-like chemical reaction + polymerization)
Composition	Resin matrix with fluoride- and calcium-releasing fillers	Modified polyacid matrix, proprietary bioactive fillers
Ion Release	High fluoride and calcium ion release	Moderate fluoride and calcium ion release
Bioactivity	Potential to form an “apatite-like” phase	Limited evidence of mineralization potential
Fracture Toughness	Relatively brittle, requires careful handling	Brittle but manageable for routine use
Wear Resistance	Acceptable for posterior restorations	Suitable for moderate mechanical demands
Durability	Good under standard clinical conditions	Adequate, with susceptibility to degradation in water
Applications	Bulk-fill restorations in posterior teeth	Versatile use in moderate caries-risk patients
Manufacturer Claims	Bioactive benefits through ion release	Enhanced fluoride release and bioactive properties

### Comparative studies

The clinical evaluation of ACT and CN has been the subject of several studies, highlighting their performance in various dental applications. Key findings from recent research (Table 6 and 7). Also, the effect of Surface Properties, Bacterial Adhesion, Thermal Aging Effects, and Energy Drink Impact on Bioactive Restorative Materials has been summarized in (Table 8).

**Table 6:** Comparative studies and clinical evaluations of CN

Comparison	Materials tested	Aim of the study	Findings	References
Fracture Resistance	Cention N, Tetric PowerFill, Beautifil II, Equia Forte HT, Surefil One	To evaluate the fracture resistance of different bioactive restorative materials in Class II MOD cavities	Cention N, Tetric PowerFill, and Beautifil II exhibited the highest fracture resistance, while Equia Forte HT and Surefil One showed the lowest	[115]
Clinical Effectiveness of IRR vs. CR	Ion-releasing restorations (IRR) vs. Composite Resin (CR)	To compare the clinical effectiveness of IRR and CR in dental restorations	No statistically significant difference was reported	[116]
Cention N vs. Equia Forte Fill	Cention N vs. High Viscosity Glass Ionomer (Equia Forte Fill)	To assess the clinical performance of Cention N compared to Equia Forte Fill over 12 months	100% success rate for Cention N at 6 and 12 months	[117]
Cention N vs. Tetric N Ceram	Cention N vs. Tetric N Ceram (nanohybrid composite resin)	To evaluate the clinical performance of Cention N in non-carious cervical lesions	Comparable for gross fracture and marginal integrity at 6 months, but Cention N had inferior initial surface characteristics	[118]
Cention N vs. GIC	Cention N vs. Glass Ionomer Cement (GIC) in Class II restorations of primary molars	To compare the clinical performance of Cention N and GIC in pediatric dentistry	Cention N showed better color match and stability at 3 months	[119]
Cention N vs. RMGIC	Cention N vs. Resin-Modified Glass Ionomer Cement (RMGIC)	To evaluate the mechanical properties of Cention N compared to RMGIC	Cention N had superior mechanical properties and reduced chair time	[120]
Cention N vs. TiO₂-enriched Cention N	Cention N vs. Titanium dioxide nanoparticle-enriched Cention N	To assess the wear resistance and durability of Cention N with TiO₂ nanoparticles	Evaluated wear resistance and durability under clinical conditions	[121]
Cention N in Pediatric Dentistry	Cention N in Class I cavities of primary molars	To assess the clinical effectiveness of Cention N in pediatric restorations	Supported its application in pediatric dentistry	[122]
Bacterial Adhesion & Surface Roughness	Tetric® N-Ceram (nanohybrid resin composite), Equia Forte™ HT Fil (glass hybrid restorative), Cention N® (alkasite)	To evaluate bacterial adhesion on two bioactive restorative materials, a glass hybrid restorative, and an alkasite with a nanohybrid resin composite as a control. Also, to assess surface roughness and its correlation with bacterial adhesion	Alkasite showed significantly lower bacterial adhesion compared to the other materials. Nanohybrid composite had the smoothest surface. No correlation was found between bacterial adhesion and surface roughness	[123]

**Table 7:** Comparative studies and clinical evaluations of ACT

Study focus	Materials compared	Aim of the study	Key findings	References
Clinical Performance in Deciduous Teeth	ACTIVA™ Bioactive Restorative	To assess the clinical performance of ACTIVA™ Bioactive Restorative in Class I and II restorations of deciduous teeth	67% of restorations remained “clinically excellent” after one year, but aesthetic properties significantly declined over time (p < 0.001)	[124]
Comparison with Equia Forte Fil	ACTIVA™ vs. Equia Forte Fil (glass hybrid)	To compare the survival rate, color match, and wear resistance of ACTIVA™ and Equia Forte Fil	Both materials had a 98% survival rate at 6 and 12 months; ACTIVA performed better in color match and occlusal contour (p < 0.001)	[125]
Comparison with Tetric EvoCeram	ACTIVA™ vs. Tetric EvoCeram (nanohybrid composite)	To evaluate clinical performance, marginal adaptation, and surface roughness over 12 months	No significant differences between materials; ACTIVA performed similarly to nanohybrid composites	[126]
Clinical & Radiographic Evaluation	ACTIVA™ BioACTIVE vs. Compomer (Dyract® eXtra)	To assess clinical and radiographic success in Class II cavities of primary molars	ACTIVA™ was not inferior to Dyract, with comparable high clinical and radiographic performance	[127]
Marginal & Internal Adaptation	Multiple restorative materials, including ACTIVA™	To evaluate the marginal and internal adaptation of restorative materials in Class II cavities	Cention Forte and Equia Forte HT showed the best marginal adaptation; ACTIVA™ was not among the top performers	[128]
Color Stability	ACTIVA™ vs. Beautifil II, Fuji II, and Filtek Z350XT	To analyze the color stability of bioactive restorative materials after immersion in staining solutions	ACTIVA™ and Filtek Z350 showed the highest color stability, while Fuji II and Beautifil II showed the least stability	[129]
Bioactive Resin Longevity & Caries Prevention	ACTIVA™ BioACTIVE vs. Conventional Composites	To determine if bioactive resin materials prevent secondary caries or improve the longevity of direct posterior restorations	No significant difference between bioactive and conventional composites in caries prevention or restoration longevity	[130]
Overall Clinical Performance & Esthetics	ACTIVA™ Bioactive Composite vs. Nanohybrid Composites	To compare ACTIVA™ Bioactive Composite with nanohybrid composites in clinical performance and esthetics	ACTIVA™ performed similarly to nanohybrid composites in marginal adaptation, marginal discoloration, and secondary caries over one year (145, 151). However, ACTIVA™ may offer superior esthetics in certain applications	[125] [126] [131]
Dentine Remineralisation	ACTIVA BioACTIVE Base/Liner, ACTIVA Presto, Predicta Bioactive Bulk vs. ProRoot MTA, MTA Angelus, Biodentine, TheraCal LC	To assess the ability of modern resin-based “bioactive” materials (RBMs) to induce dentine remineralisation via mineral deposition and compare them with calcium silicate cements (CSMs)	ProRoot MTA, MTA Angelus, Biodentine, and TheraCal LC showed significant mineral precipitation and filled the gap between material and dentine. RBMs showed only slight mineral precipitation and were unable to remineralise the dentine-material interface. CSMs were the only materials that induced a reparative process at the interface	[132]
Microleakage in Primary Teeth	ACTIVA™ Kids BioActive-Restorative vs. Zirconomer Improved, Beautifil II, Vitremer™	To evaluate and compare the microleakage of bioactive restorative materials in Class V cavities of primary teeth	The least microleakage was observed in Vitremer™, followed by Beautifil II, Zirconomer Improved, and ACTIVA™ Kids BioActive. ACTIVA™ exhibited the highest microleakage	[133]
Clinical Performance of Ion-Releasing Liners (NCT05470959)	Activa Bioactive Base/Liner vs. Riva Light Cure (resin-modified glass ionomer)	To evaluate the impact of using ion-releasing liners on the 3-year clinical performance of posterior resin composite restorations after selective caries excavation with polymer burs	The success rates were 100% for all resin composite restorations, regardless of whether they were lined with Activa Bioactive Base/Liner or Riva Light Cure. No significant differences between the two liner materials in clinical performance (p > 0.05) over 3 years	[134]
Color Change of Bioactive Materials after Whitening Agents	Filtek Z250, Equa Forte HT, Activa Bioactive Restorative	To examine the color changes of bioactive materials after the application of different office whitening agents	Activa Bioactive Restorative showed the highest color change (ΔE00) after whitening treatment. Z250 showed the least color change. FGM whitening agent caused the highest color change after coffee immersion	[135]
Shear Bond Strength to Dentin	Fuji II RMGIC (with and without chitosan), ACTIVA Bio-ACTIVE Restorative	To evaluate the impact of adding chitosan to Fuji II resin-modified glass ionomer cement (RMGIC) on shear bond strength to dentin and compare it with ACTIVA Bio-ACTIVE Restorative	The addition of chitosan to Fuji II RMGIC resulted in a significant reduction in shear bond strength to dentin. Both Fuji II and ACTIVA Bio-ACTIVE Restorative exhibited favorable bond strength compared to the chitosan-modified Fuji II	[136]
Nanohardness, Elastic Modulus, and Surface Roughness	EQUIA Forte (GC), Dyract XP (Dentsply), Activa BioACTIVE (Pulpdent)	To evaluate the nanohardness, elastic modulus, and surface roughness of fluoride-releasing tooth-colored restorative materials after immersion in acidic beverages	Activa BioACTIVE exhibited the highest initial nanohardness. After immersion, it retained the highest elastic modulus, while Dyract XP showed the lowest surface roughness. Acidic beverages negatively impacted the properties of all materials	[137]
Ion-Releasing Capability & Micromorphological Patterns	Giomer (Beautifil II), Ion-Releasing Composite (Activa Presto), Resin-Modified Glass Ionomer (Riva Light Cure)	To evaluate and compare the ion-releasing capability of three restorative systems at the restoration/tooth interface using elemental analysis, and examine micromorphological patterns of the interface	Significant differences were found in phosphorus and calcium levels. RMGI had the highest phosphorus levels initially, while the ion-releasing composite had the highest calcium levels. The restorative materials exhibited good micromorphological adaptation to the tooth substrate	[138]

**Table 8:** The effect of surface properties, bacterial adhesion, thermal aging effects, and energy drink impact on bioactive restorative materials

Study focus	Materials compared	Aim of the study	Key findings	References
Effect of Radiotherapy on Adhesive Interface	Fuji II LC, EQUIA Forte HT, Beautifil II, Cention N, Activa Bioactive Restorative, Filtek Z250	To analyze defects in the adhesive interface formed with five bioactive dental materials and caries-affected dentin concerning the timing of radiotherapy	RT2 caused significantly higher adhesive defects for Filtek Z250 and Activa Bioactive Restorative. No significant differences were found for RT1. High-viscosity glass ionomer hybrid cement (EQUIA Forte HT) and giomer (Beautifil II) showed favorable results compared to dual-cure bioactive bulk-fill composite and alkasite restorations	[139]
Effect of Thermal Aging on Surface Properties and Ion Release	Cention N, ACTIVA BioActive Restorative, Equia Forte HT Fil, Glass Fill (GCP), Fuji II LC	To evaluate the effect of thermal aging on surface microhardness, surface chemical composition, and ion release	Cention N had the highest microhardness before thermal aging. The fluoride ion ratio decreased in all groups except Cention N after thermal aging. ACTIVA BioActive Restorative had a microporous surface after aging. Thermal aging affected microhardness, surface properties, and ion release. Alkasite bioactive materials showed better mechanical properties than other materials	[140]
Effect of Energy Drinks on Bioactive Materials	Charisma Diamond One, Activa™ BioActive Restorative, Activa™ Presto™, Equia Forte HT Fil	To compare the effects of energy drinks on surface roughness, weight loss, and color change of various bioactive materials	Powerade and Monster roughened the surface of all materials. Burn affected all materials except Activa BioActive. Significant weight loss was observed in Equia Forte HT Fil after immersion in all energy drinks. Color change (ΔE00) was greatest in the Burn and Monster groups, except for Equia Forte HT Fil	(141)

## Challenges and future directions

The development and application of bioactive restorative materials face several challenges that need to be addressed to fully realize their potential, such as the lack of standardized testing protocols for bioactivity. Enhancing the mechanical strength, wear resistance, and fracture toughness of bioactive materials is essential for expanding their clinical applications, particularly in stress-bearing areas. Incorporating reinforcing fillers, optimizing the matrix composition, and exploring novel material combinations are potential strategies for improving mechanical properties. Limited long-term clinical data raise the need for conducting clinical trials with extended follow-up periods is essential for evaluating their durability, success rates, and potential complications.

## Conclusion

Bioactive restorative materials represent a significant advancement in modern dentistry, offering the potential to actively promote oral health and improve patient outcomes. These materials can stimulate tissue repair, remineralization, and reduce secondary caries. However, a critical and evidence-based approach is essential when selecting and using bioactive restorative materials.

Continued research and development are needed to address the existing challenges and fully realize the potential of bioactive materials. By developing standardized assessment methods, improving mechanical properties, conducting long-term clinical trials, and exploring novel materials, we can pave the way for a new era of restorative dentistry focused on bioactivity, regeneration, and long-term oral health. The integration of bioactive materials into dental education and clinical practice guidelines is also crucial for promoting their appropriate and effective use.

## Data Availability

The data used to support the findings of this study are included within the article.
